# Beyond the baseline: quantification of two phosphatidylethanol homologues in whole blood by LC–MS-MS and retrospective data analysis from a National Reference Laboratory

**DOI:** 10.1093/jat/bkae100

**Published:** 2025-01-13

**Authors:** Nicole J Mathewson, Nkemakonam C Okoye, Heather A Nelson, Vrajesh Pandya, Chad Moore, Kamisha L Johnson-Davis

**Affiliations:** Department of Pathology, University of Utah Health, Salt Lake City, UT 84108, USA; ARUP Laboratories, ARUP Institute for Clinical and Experimental Pathology, Salt Lake City, UT 84108, USA; Department of Pathology and Laboratory Medicine, Northwell Health, New Hyde Park, NY 11042, USA; Department of Pathology, University of Utah Health, Salt Lake City, UT 84108, USA; ARUP Laboratories, ARUP Institute for Clinical and Experimental Pathology, Salt Lake City, UT 84108, USA; Department of Pathology, University of Utah Health, Salt Lake City, UT 84108, USA; ARUP Laboratories, ARUP Institute for Clinical and Experimental Pathology, Salt Lake City, UT 84108, USA; Sports Medicine Research and Testing Laboratory, Salt Lake City, UT 84108, USA; Department of Pathology, University of Utah Health, Salt Lake City, UT 84108, USA; ARUP Laboratories, ARUP Institute for Clinical and Experimental Pathology, Salt Lake City, UT 84108, USA

## Abstract

Alcohol is the most abused substance in Western society, resulting in major economic losses and negative health consequences. Therefore, there is a need for a selective and robust detection method for alcohol consumption in various clinical and forensic settings. This study aimed to validate a mass spectrometry method for quantifying phosphatidylethanol (PEth) and perform retrospective data analysis from the patient population of a national reference laboratory. Quantification of PEth in whole blood was accomplished using an LC–MS-MS assay. Isotopically labeled internal standard for the two PEth homologues was added to the whole-blood specimen, followed by protein precipitation with a mixture of acetonitrile and isopropyl alcohol. After centrifugation, an aliquot of the supernatant was buffered with ammonium acetate before LC–MS–MS analysis on an Agilent 6470 triple quadrupole mass spectrometer coupled to an Agilent 1260 Infinity II LC system. This LC–MS-MS assay was validated for clinical use in accordance with Clinical & Laboratory Standards Institute guidelines. The analytical measurement range, 10–2000 ng/mL, was linear with *R*^2^ of 0.999. The within-run and total imprecision was < 5% CV for the low (20 ng/mL), medium (200 ng/mL), and high QC (1000 ng/mL). Results from accuracy and method comparison experiments met the bias criteria of ±15%. Retrospective data analysis showed ∼27% of patients had PEth concentrations <20 ng/mL. Males and females had similar positivity rates for PEth and the positivity rate of women of reproductive age (15–44 years old) was 35% in comparison to 25% in women 45–89 years old. This study’s LC–MS-MS method showed acceptable analytical performance in quantifying PEth as a sensitive and specific biomarker for evaluating alcohol consumption. Results from this study may provide an opportunity to educate women of reproductive age on drinking during pregnancy and the long-term effects of alcohol use.

## Introduction

Alcohol use disorder remains a significant health challenge in the USA. According to the 2023 National Survey on Drug Use and Health, approximately 134.7 million Americans aged ≥12 years, representing >40% of the population, reported current alcohol use, defined as any alcohol consumption within the past 30 days[1]. Among them, about 61.4 million individuals, or roughly 45% of alcohol users, engaged in binge drinking, defined as consuming five or more drinks for males and four or more drinks for females on a single day within the past 30 days [1]. Additionally, over 16 million Americans were identified as heavy alcohol users, defined as those who binge drink for ≥5 days within the past 30 days [1].

Assessing alcohol exposure is imperative for detecting driving under the influence and supporting emergency departments in managing cases of alcohol intoxication or alcohol-induced coma. Alcohol biomarker testing also plays a crucial role in monitoring adherence to pain management regimens, compliance with drug and alcohol abstinence programs, and prequalification screening for organ transplantation. In addition, it can aid in identifying prenatal alcohol exposure in neonates to facilitate early intervention. Approximately 9.8% of pregnant women aged 15–44 years in the USA reported alcohol use in the past month, with 4.3% of them reporting binge drinking during the same period [1]. Prenatal alcohol exposure during the first trimester of pregnancy is considered highly detrimental to fetal development [2]. A study supported by the National Institute on Alcohol Abuse and Alcoholism (NIAAA) of more than 6000 children in first grade across four US communities estimated that, as many as 1–5% of first-grade children have fetal alcohol spectrum disorder (FASD) [3]. Currently, pregnant women are typically screened for alcohol consumption through subjective self-report methods, which may underestimate actual usage [4]. Therefore, a reliable biomarker to detect and monitor alcohol use is necessary.

Blood alcohol concentration (BAC) in serum/plasma or in breath is often used to assess acute alcohol consumption; however, BAC has a narrow detection window (< 12 h) [5–7]. The nonoxidative ethanol metabolites, ethyl glucuronide, and ethyl sulfate, are direct biomarkers of ethanol exposure with a detection window of 2–5 days in urine [8.] Indirect biomarkers for alcohol exposures, such as liver enzymes, gamma glutamyl transferase (GGT), aspartate aminotransferase (AST), and alanine aminotransferase (ALT) can increase as a result of heavy alcohol use; however, these liver enzymes can also increase due to liver injury or disease, unrelated to alcohol use disorders; therefore, these enzymes are not specific for alcohol consumption [5–7]. Carbohydrate-deficient transferrin (CDT) is a specific and indirect biomarker for heavy drinking (approximately four standard drinks) over several weeks. CDT is formed under conditions of heavy alcohol consumption, which will decrease the glycosylation of transferrin to produce transferrin that is free of sialic acid. CDT will reduce to normal concentrations after ∼14 days of alcohol cessation. However, CDT is not a sensitive marker to detect light to moderate alcohol users [7].

Consequently, there is a clinical need for a direct alcohol biomarker that is highly specific and has a longer window of detection for acute and chronic exposure. Phosphatidylethanol (PEth) is a direct ethanol marker in the blood that detects acute and chronic heavy drinking with high specificity. PEth is a group of phospholipids that can form in tissues such as the brain, liver, platelets, lymphocytes, and red blood cells (RBCs) [9]. PEth can form on the membrane of RBC in the presence of ethanol and the enzyme phospholipase D (PLD) [10–12]. PLD has ∼1000-fold greater affinity for ethanol than water; therefore, in the presence of ethanol, PLD catalyzes a transphosphatidylation reaction of phosphatidylcholine to form PEth homologs [11, 13]. In the absence of ethanol in the blood, PLD hydrolyzes phosphatidylcholine to produce phosphatidic acid and choline [10]. Once produced, PEth is incorporated into erythrocyte cell membranes and can be detected following ethanol consumption, and the amount of formation is dependent on the amount of alcohol consumed and frequency [12]. PEth is detectable after drinking 1–2 alcoholic beverages, and it has a half-life of 4–10 days after ethanol use [12, 14, 15]. The half-life of PEth in the blood is prolonged in RBCs, in comparison to other tissues because the cells do not contain the enzyme, phosphatidylcholine phospholipase C, which degrades PEth. The window of detection can span weeks to months with heavy and/or chronic alcohol consumption. There are 48 known homologues of PEth that have been identified and these homologues are distinguished by the number of carbons and double bonds in the fatty acid chains [16]. PEth homologues have a common nonpolar phosphoethanol head group with two fatty acid moieties. 1-Palmitoyl-2-oleoyl-phosphatidylethanol (PEth 16:0/18:1, POPEth) and 1-palmitoyl-2-linoleoyl-phosphatidylethanol (PEth 16:0/18:2, PLPEth) are the predominant PEth homologues in whole blood and account for 37–46% and 26–28% of the total PEth homologues, respectively [13, 15].

The homologues of PEth are analyzed by mass spectrometry [13, 17–21] and may be used to support patient care to prequalify individuals for organ transplantation [22–26], to assess drinking during pregnancy [4, 27–35], to assess neonatal alcohol exposure [34, 36, 37], and to monitor compliance for alcohol abstinence [38–41]. The concentration of PEth in whole blood can be used to characterize patients as chronic or heavy drinkers, social/light drinkers, and identify relapse drinking after alcohol abstinence [13, 42–46].

The purpose of this study was to develop and validate a method to quantify two homologues for PEth by LC–MS-MS and perform retrospective data analysis to evaluate the clinical utility of measuring two homologues to detect alcohol exposure and to assess the correlation of PEth with paired testing for additional ethanol biomarkers, CDT, and EtG/EtS. To investigate alcohol exposure in women of reproductive age at the University of Utah Health, residual whole-blood patient samples were collected from the OB/GYN and non-OB/GYN clinical units, to measure PEth.

## Materials

### Regents

PEth 16:0/18:1 (POPEth) and 16:0/18:2 (PLPEth) standards were purchased from Cerilliant (Round Rock, TX, USA) and the internal standards POPEth-d_5_ and PLPEth-d_5_ were purchased from Echelon Biosciences (Salt Lake City, UT, USA). HPLC-grade solvents for methanol, acetonitrile, and isopropyl alcohol were purchased from VWR Scientific (Radnor, PA, USA) and ammonium acetate was purchased from Sigma-Aldrich (St. Louis, MO) to make mobile phase in clinical laboratory reagent water (CLRW). Blank human whole blood was drawn from donors and collected in tubes with anticoagulants (K2 EDTA, K3 EDTA, lithium heparin, or potassium oxalate are acceptable). The blood from donors was prescreened to ensure the absence of POPEth and PLPEth before preparing calibrators and quality control (QC) samples.

## Methods

### Institutional review board

University of Utah Institutional Review Board (IRB Protocol #00082990, #00140204) approved this retrospective analysis of clinical samples from human subjects. Retrospective patient data was de-identified from ARUP Laboratories, using samples from the PEth, EtG/EtS, and CDT assays. To compare the agreement of alcohol biomarkers EtG, EtS, and CDT with PEth, paired patient specimens were used from clients who ordered more than one alcohol biomarker assay on the same patient and day of specimen collection. For the PEth assay, the age range was 1–101 years (total *N* = 235 504; males *N* = 142 713, females, *N* = 92 841). Retrospective data were also used to evaluate the positivity rates of PEth in males, females (15–89 years old), and women of reproductive age (15–44 years old). Patients with non-numeric results were excluded from the analysis (“Not Applicable,” “Present,” or “NULL”). To evaluate alcohol use in females of reproductive age at the University of Utah Health, residual whole-blood specimens from females aged 15–44 years old were collected, within the timeframe of 2 months, from the OB/GYN (*N* = 209) and non-OB/GYN (*N* = 132) clinical units in the greater Salt Lake City, Utah region, then analyzed for PEth at ARUP Laboratories. The OB/GYN clinical units included general OB/GYN, labor and delivery, emergency OB/GYN services, maternal newborn care, and women’s special care. The non-OB/GYN clinical units included internal medicine, neurology, emergency department, cardiovascular, surgical transplant, intensive care, and family practice. Data analysis was performed using Microsoft Excel.

#### Patient sample preparation for PEth analysis

Anticoagulated whole blood from patient specimens, calibrators, and QC samples were equilibrated at room temperature while mixing on a rocker for 30 min. The calibrator concentrations for POPEth and PLPEth were 10, 50, 400, 800, 1400, and 2000 ng/mL and the QC concentrations were 20, 200, and 1000 ng/mL. A total of 50 µl of internal standards (POPEth-d_5_ and PLPEth-d_5_, each at 600 ng/mL) was added into 100 µl aliquots of calibrators, QC, and whole-blood patient samples in a 96-well plate, 2.2 ml (Agilent Technologies, Santa Clara, CA). The plate was sealed with a cap mat and vortexed for 5 min on a multitube vortexer. The cap mat was removed and protein precipitation was achieved by adding 500 µl of 10% (v/v) isopropanol (IPA)/acetonitrile (ACN) 90% (v/v) solution to each sample. The 96-well plate was resealed with a cap mat and mixed for 10 min on a multitube vortexer. The plate was centrifuged for 5 min at 3500 rpm (∼3200 rcf). Upon centrifugation, 200 µl of the supernatant extract was transferred to another 96-well plate using a VIAFLO96 liquid handler (Integra Biosciences, Hudson, NH, USA) and buffered with 15 mM ammonium acetate in CLRW (pH unadjusted). The plate was mixed and then centrifuged for 1 min (∼1000 rcf).

#### Sample analysis by LC–MS-MS

Sample extracts were analyzed by LC–MS-MS using an Agilent 6470 triple quadrupole mass spectrometer coupled to an Agilent LC system equipped with two 1260 Infinity II binary HPLC pumps, a 1260 Infinity II autosampler, and a 1260 Infinity II thermostat column compartment. The mass spectrometry method was performed using negative ion electrospray with multiple reaction monitoring acquisition. Chromatographic separation was achieved using two Phenomenex Luna Omega Polar C18 columns (100A, 1.6 µm 2.1 × 50 mm) (Torrance, CA, USA) maintained at 60°C on a 0.5 ml/min gradient. Automated alternating column regeneration was used to increase the chromatographic throughput by running two columns simultaneously, where LC pump 1 performs chromatographic separation on one column, while LC pump 2 washes and regenerates the other column. The two LC columns are alternated between pumps 1 and 2 with each sample injection. The mobile-phase program for chromatographic separation (pump 1) consisted of 5 mM ammonium acetate in 30/70 H_2_O/ACN (mobile phase A) and 5 mM ammonium acetate in 30/70 IPA/ACN (mobile phase B) for LC pump 1 (Table 1). For column washing/regeneration (pump 2), the column was washed with 100% mobile phase A consisting of 5 mM ammonium acetate in 80/18/2 IPA/methanol/H_2_O and then regenerated with 100% mobile phase B consisting of 5 mM ammonium acetate in 70/27/3 ACN/H_2_O/IPA (i.e. the initial mobile-phase condition of pump 1) (Table 1). The needle wash was LC–MS grade methanol. The injection volume was 20 µl with a chromatographic run time of 3.5 min. The retention time was 1.4 min for POPEth and POPEth-d_5_ and 1.6 min for PLPEth and PLPEth-d_5_. The mass spectrometer settings are listed in Table 2 and the mass transitions for POPEth and PLPEth are listed in Table 3. The total number of patient samples analyzed was 235 504, (males = 142 713, females = 92 841) and the age range was 1–101 years. The POPEth results were interpreted as: < 10 ng/mL = not detected, 10 to < 20 ng/mL = abstinence or light alcohol consumption, 20–200 ng/mL = moderate alcohol consumption, > 200 ng/mL = heavy alcohol consumption or chronic use [42, 47]. There are no established result interpretation ranges for PLPEth.

**Table 1.** Mobile-phase gradient

		Pump 1			Pump 2		
Step	Time (min)	Flow rate (ml/min)	A (%)	B (%)	Flow rate (ml/min)	A (%)	B (%)
1	0	0.5	90	10	0.45	100	0
2	0.2	0.5	90	10			
3	0.21	0.5	40	60			
4	1.5				0.45	100	
5	1.51				0.5	0	100
6	2	0.5	5	95			
7	2.01	0.5	90	10			
8	3.5	0.5	90	10	0.5	0	100

**Table 2.** Mass spectrometer settings

Gas temp (°C)	325
Gas flow (l/min)	9
Nebulizer (psi)	25
Sheath gas temp (°C)	400
Sheath gas flow (l/min)	12
Capillary neg (nA)	−3500
Nozzle (V)	0
Delta EMV (−)	200
Q1 resolution	Unit
Q3 resolution	Unit

**Table 3.** PEth LC–MS-MS mass transitions

	Analyte	Q1 Mass (Da)	Q3 Mass (Da)	Fragmentor	Dwell	Collision energy	Cell accelerator voltage
**1**	PLPEth 1	699.5	279.2	192	25	29	4
**2**	PLPEth 2	699.5	255.2	192	25	33	4
**3**	POPEth 1	701.5	281.2	192	25	34	4
**4**	POPEth 2	701.5	255.2	192	25	38	4
**5**	PLPEth-d_5_ 1	704.5	279.2	192	25	33	4
**6**	PLPEth-d_5_ 2	704.5	255.2	192	25	41	4
**7**	POPEth-d_5_ 1	706.5	281.2	192	25	34	4
**8**	POPEth-d_5_ 2	706.5	255.2	192	25	38	4

### Carbohydrate-deficient transferrin

CDT was measured on the Sebia MINICAP System. In this method, serum is diluted on-board with a specific sample diluent included in the kit. The diluted specimen is injected at the anodic end of a capillary and proteins are separated by high-voltage electrophoresis in alkaline buffer (pH 8.8). The transferrin glycoforms are detected at the cathodic end of the capillary by measuring their absorbance at 200 nm. Transferrin isoforms are detected in the following order: asialotransferrin, disalotransferrin, trisialotransferrin, tetrasialotransferrin, and pentasialotransferrin. Relative quantification of individual transferrin isoforms is performed and reported as a percentage of the asialotransferrin and disalotransferrin over the sum of all glycoforms. CDT ≤1.3% is a normal result, while CDT ≥1.7% is considered positive, consistent with chronic alcohol abuse.

### Ethyl glucuronide and ethyl sulfate

Urine specimens were analyzed for ethyl glucuronide and ethyl sulfate by LC–MS. [48]

## Results

Analytical validation of the LC–MS-MS method was performed in whole blood for accuracy, linearity, imprecision, sensitivity, specificity, and interference, using CLSI guidance document C62-A, LC–MS methods.

### Accuracy/method comparison

One hundred patient samples were analyzed and compared to an external reference laboratory for POPEth. For PLPEth, blank whole blood was fortified with concentrations that spanned the analytical measurement range 10–2000 ng/mL. Method comparison studies showed excellent correlation by Deming regression analysis (*y* = 1.003*x*−21.4; *R* = 0.9745) for POPEth. For PLPEth, blinded spikes samples, with a minimum of 20 different concentrations were tested with 5 replicates per concentration over ≥ 3 days and the Deming regression analysis was (*y* = 0.933*x* + 9.41; *R* = 0.9945). Accuracy was within ±15% from target concentration.

### Linearity

The six calibrators (10, 50, 400, 800, 1400, and 2000 ng/mL) for POPEth and PLPEth were analyzed in human whole blood with EDTA anticoagulant, with ≥ 3 replicates over 2 days. The linear regression for POPEth was *y* = 1.02*x*−14.87 and PLPEth was *y* = 1.01*x*−9.55, with *R*  [2] of 0.999 for both homologues.

### Imprecision

QC concentrations of 20, 200, and 1000 ng/mL for POPEth and PLPEth were analyzed in human whole blood with EDTA anticoagulant, in replicates of 5 over 4 days. Total imprecision was < 10% CV.

### Sensitivity

The lower limit of quantifications (LLOQ) were analyzed for POPEth and PLPEth at 10 ng/mL in human whole blood with EDTA anticoagulant, in 5 replicates over 4 days. The signal-to-noise ratio at the lower limit of quantitation (10 ng/mL) was 63:1. Imprecision at the LLOQ was < 8% CV for both PLPEth and POPEth.

### Analytical specificity/interfering compounds

Fifty drugs/metabolites were spiked into whole blood with POPEth and PLPEth at 10 ng/mL, with 3 replications for 1 day. Hemoglobin (1800 mg/dL), bilirubin (7.25 mg/dL), and triglyceride (4700 mg/dL) were spiked into whole blood with POPEth and PLPEth at 10 ng/mL, with 3 replications for 1 day. Accuracy was ≤ ±10% and imprecision were ≤ 10%.

### Retrospective data analysis

**Figure 1.**
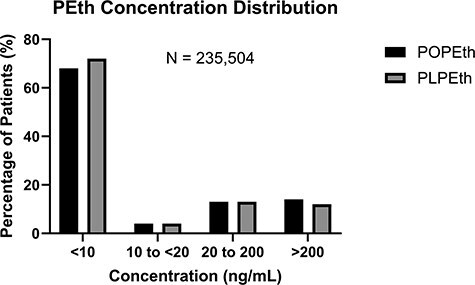
PEth concentration distribution and positivity rates.

**Figure 2.**
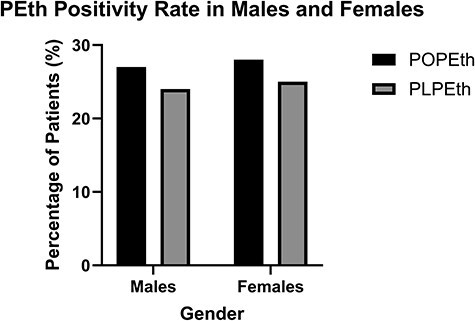
PEth positivity rates in males and females.

**Figure 3.**
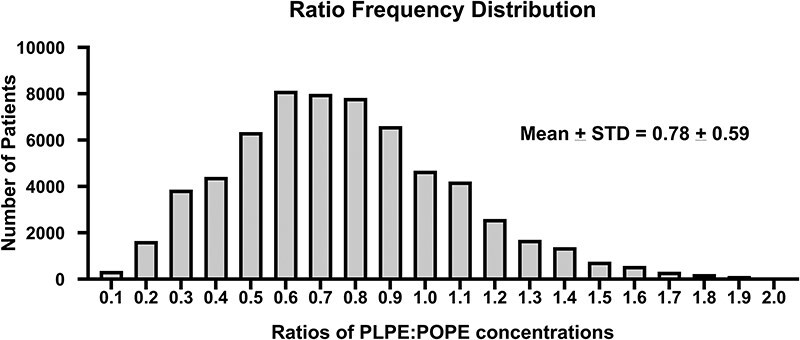
A histogram of the PLPEth:POPEth ratio was created using patients with PLPEth and POPEth concentration ≥ 10 ng/m, using a bin width of 0.1.

The PEth LC–MS-MS assay was used to assess alcohol exposure from the patient population tested at ARUP Laboratories (*N* = 235 504). Out of 235 504 patient samples, POPEth and PLPEth were below the limit of quantitation (10 ng/mL) in 68% (*N*= 161 143) and 72% (*N* = 169 646) of whole-blood specimens submitted for routine PEth testing, respectively. Four percent of patients had concentrations between 10 and < 20 ng/mL for POPEth (*N* = 9990) and PLPEth (*N* = 8816), suggesting abstinence or light alcohol consumption. Notably, 3.6% of patients in the data set had POPEth concentrations ≥ 10 ng/mL, with PLPEth concentrations < 10 ng/mL. Based on current consensus interpretative guidelines [42, 47], Fig. 1 shows that 13% of individuals met criteria for moderate alcohol consumption (POPEth 20–200 ng/mL, *N* = 31 738; PLPEth 20–200 ng/mL, *N* = 29 836) and 14% of met criteria for heavy alcohol consumption (POPEth >200 ng/mL, *N* = 32 584; 12% PLPEth > 200 ng/mL, *N* = 27 254). The PEth concentrations of both homologues were similar in concentration for each group with a direct correlation (Spearman *r* = 0.940). There was no discernable correlation between age and concentration of PEth homologues. Likewise, no significant gender difference in PEth concentration was observed. Fig 2 demonstrates that the positivity rate of PEth in males and females were similar (27–28%). The concentration ratio of PLPEth to POPEth was evaluated in patients (*N* = 63 985) that were positive for both homologues at concentrations ≥ 10 ng/mL. A histogram was created using the bin width of 0.1 increments, the PLPEth:POPEth ratios ranged from 0 to 2.0 (Fig. 3). The mean ratio of PLPEth:POPEth was 0.78 with a standard deviation of ± 0.59 (mean ± STD: 0.19–1.37), (Fig. 3).

### Paired patients orders for EtG/EtS and PEth

The large data set was used to identify patients in which whole blood and urine specimens were collected for paired orders of EtG/EtS and PEth (Table 4). Noteworthy, patients were excluded from analysis if results lacked a quantitative result for EtG or EtS due to analytical interference (*n* = 79, EtG, *n* = 53, EtS). Consequently, PEth was compared separately to EtG and EtS. In this population, the negative agreement was 79.1% for patients with EtG concentrations reported as < 100 ng/mL and POPEth reported as < 10 ng/mL. PEth was detected in 21% of patient samples when EtG was not detected; however, in patients with EtG concentrations > 100 ng/mL, PEth was not detected in 15.3% of samples (Table 4). The negative agreement for patients with EtS concentrations reported as < 100 ng/mL and POPEth reported as < 10 ng/mL was 71.4%. PEth was detected in 28.5% of patient samples when EtS was not detected; however, PEth was not detected in 12.5% of patients with EtS concentrations > 100 ng/mL (Table 4).

**Table 4.** Results of paired testing for EtG/EtS or CDT with PEth

	POPEth				Total
EtG (ng/mL)	Not detected	Light	Moderate	Heavy	*N* = 2082
<100	672 (79.1%)	56 (6.6%)	90 (10.6%)	31 (3.7%)	849 (40.8%)
100–10 000	106 (15.1%)	28 (4.0%)	207 (29.4%)	362 (51.5%)	703 (33.8%)
>10 000	1 (0.2%)	0	97 (18.3%)	432 (81.5%)	530 (25.4%)
	**POPEth**				**Total**
**EtS (ng/mL)**	Not detected	Light	Moderate	Heavy	***N* = 2108**
<100	705 (71.4%)	61 (6.2%)	118 (11.9%)	103 (10.4%)	987 (46.8%)
100–10 000	88 (12.2%)	24 (3.3%)	219 (30.2%)	393 (54.3%)	724 (34.3%)
>10 000	1 (0.3%)	0	62 (15.6%)	334 (84.1%)	397 (18.8%)
	**POPEth**				**Total**
**CDT**	Not detected	Light	Moderate	Heavy	***N* = 260**
Normal	134 (62.9%)	13 (6.1%)	42 (19.7%)	24 (11.3%)	213 (81.9%)
Inconclusive	1 (6.2%)	0	5 (31.3%)	10 (62.5%)	16 (6.2%)
Elevated	3 (9.7%)	0	3 (9.7%)	25 (80.6%)	31 (11.9%)

### Comparison of CDT and PEth

The PEth dataset was also used to ascertain patients with paired orders for PEth and CDT. Out of 406 paired orders on the same patient, 260 pairs had a result for both CDT and PEth and were included in subsequent analysis. The 146 paired orders excluded from analysis did not have a result for CDT due to analytical interference (*n* = 130) or presence of an unusual transferrin isoform(s) (*n* = 16). In samples in which CDT was ≤ 1.3%, POPEth was not detected (<10 ng/mL) in 63% of paired patient orders (Table 4). CDT was elevated (≥1.7%) in 25 patients (80.6%) with POPEth >200 ng/mL, 3 patients (9.7%) with POPEth in the moderate alcohol consumption range (POPEth 20–200 ng/mL), and in 3 patients (9.7%) who had undetectable POPEth. An inconclusive CDT result (1.4–1.6%) was obtained in 10 patients (62.5%) with heavy alcohol consumption by PEth (POPEth >200 ng/mL), 5 patients (31.3%) with moderate consumption by PEth (POPEth 20–200 ng/mL), and in 1 patient (6.2%) with undetectable PEth. Most discrepancies observed were in patients with a normal CDT result, but positive PEth result (*n* = 79). 24 patients (9%) with a normal CDT had a POPEth >200 ng/mL, 42 fell in the moderate alcohol consumption range (POPEth 20–200 ng/mL), and 13 in the light alcohol consumption range (10 to < 20 ng/mL) (Table 5).

### Women of reproductive age

**Figure 4.**
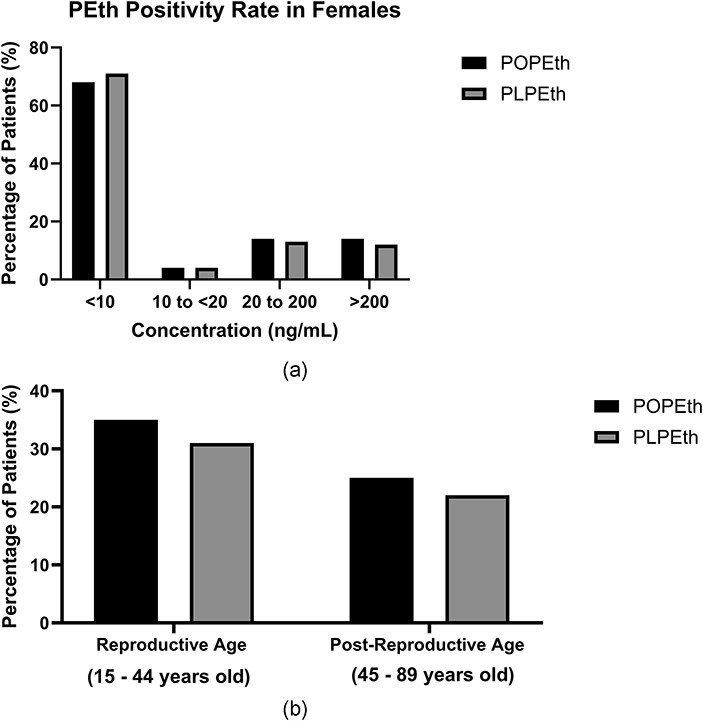
Retrospective data analysis illustrating the positivity rates of PEth in females. (a) PEth concentration distribution in females aged 15–89 years old. The POPEth concentration indicates the level of alcohol consumption, ranging from abstinence to heavy alcohol use. (b) Positivity rates of PEth in reproductive and postreproductive aged women.

The data set of 235 504 patients from the PEth assay was used to evaluate the positivity rates of alcohol exposure in females (15–89 years, *N* = 92 726) and in women of reproductive age (15–44 years). The positivity rate was determined by dividing the number of positive PEth results (≥20 ng/mL) by the total number of results, yielding a positivity rate of PEth in females (15–89 years old) to 28% for POPEth and 25% for PLPEth. Furthermore, the distribution of PEth concentrations in females illustrated that 68% of the results were <10 ng/mL (not detected), 4% were 10 to < 20 ng/mL (abstinence or light alcohol consumption), 14% were 20–200 ng/mL (moderate alcohol consumption), and 14% were > 200 ng/mL (heavy alcohol consumption or chronic use) (Fig. 4a). The positivity rate of PEth in women of reproductive age (15–44 years) was 35% (*N* = 32 454) for POPEth and 31% (*N* = 28 745) for PLPEth (Fig. 4b). The positivity rate of PEth in women of postreproductive age (45–89 years) was 25% (*N* = 28 181) for POPEth and 22% (*N* = 20 399) for PLPEth (Fig. 4b).

### University of Utah Health

**Figure 5.**
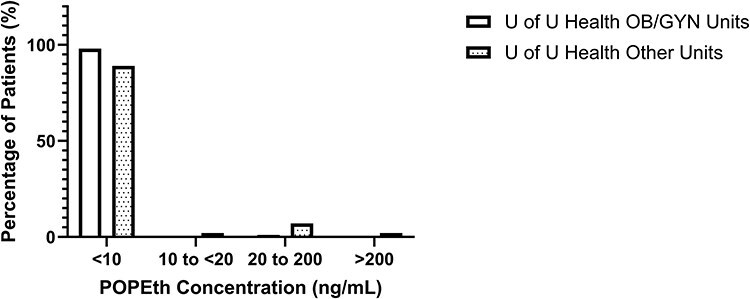
PEth concentration distribution in University of Utah Health OB/GYN and University of Utah Health non-OB/GYN clinical units from laboratory investigation.

Residual whole-blood specimens collected from women of reproductive age at the University of Utah Health, revealed a significantly lower PEth positivity rate in the OB/GYN clinics (1%, *N* = 209) and other non-OB/GYN (9%, *N* = 132) clinical units when compared to the PEth retrospective data analysis in the large data set for women of reproductive age from the state of Utah (27%) and women of reproductive age in other states combined (35%). Furthermore, 98% and 89% of the residual samples collected from the Utah OB/GYN and other clinical units, respectively, had POPEth and PLPEth concentrations of <10 ng/mL, which indicates that PEth was not detected (Fig. 5).

## Discussion

PEth has emerged as an important biomarker for monitoring alcohol exposure [22, 49]. The interpretative guidelines for PEth concentrations provide a useful framework for interpreting results in clinical assessments of alcohol use disorders and monitoring alcohol abstinence, especially in transplant candidates and patients. It is estimated that up to 50% of patients that received a liver transplant from alcohol liver disease, consume alcohol, within 5–10 years post-transplant [50–52]. Routine monitoring of alcohol exposure pre- and post-transplant is imperative to prevent or identify relapse, in order to improve post-transplant survival [52]. Interestingly, our retrospective data analysis revealed that 4% of patients had PEth (POPEth and PLPEth) concentrations between 10 and < 20 ng/mL, 27% had PEth concentrations ≥20 ng/mL, and 69% of the specimens did not have PEth detected, suggesting the patients being monitored for alcohol exposure, are abstaining from alcohol in our patient population. Males and females in this population had similar positivity rates, and PEth concentrations were not significantly different, which supports previous clinical studies [53, 54]. Our retrospective data support previous studies demonstrating the high sensitivity and specificity of detecting alcohol exposure using PEth, compared to EtG/EtS and CDT [55–59]. Moreover, PEth was useful in paired ordered specimens to detect alcohol exposure when CDT was inconclusive or had an assay interference.

The observed direct correlation between POPEth and PLPEth concentrations supports the specificity of our assay to monitor PEth for alcohol consumption for clinical testing using a robust LC–MS-MS method. POPEth and PLPEth have different rates of formation, elimination, and half-lives, and previous clinical studies have demonstrated that PLPEth can form at a faster rate in response to alcohol exposure and has a faster elimination in comparison to POPEth during alcohol abstinence [45, 57, 60–62]. Of note, a study from Varga *et al*. (1998) identified that patients with liver disease had a smaller PLPEth to POPEth ratio when alcohol was consumed within a week before specimen collection [63].. In this study, the ratios of PLPEth to POPEth were assessed, and the ratios spanned from 0.1 to 2.0, with the mean ± STD of 0.78 ± 0.59. Monitoring both PEth homologue concentrations over time could be used to evaluate the timeframe since the last alcohol exposure and improve sensitivity to evaluate alcohol abstinence and identify acute alcohol exposure [15, 64]. Interestingly, Naik *et al*. (2020) performed experiments to analyze PEth homologues in pregnant rats that were chronically exposed to alcohol and found that PEth 16:0/18:2 was more abundant in maternal and fetal blood than PEth 16:0/18:1 and 16:0/20:4, suggesting that monitoring more than one PEth homologue can improve sensitivity for detecting alcohol exposure [65]. Moreover, incorporating PLPEth in our analytical method was also used as a secondary PEth homologue to support the POPEth result and reassure clinicians that a false positive did not occur.

The laboratory investigation provided additional insight into PEth positivity rates among women of reproductive age in our patient population and for a small number of patients at the University of Utah Health. Self-report studies for alcohol use demonstrate that pregnant women tend to underreport their consumption [34]. PEth has shown to be a highly specific biomarker to identify individuals who are consuming alcohol during pregnancy and may be more reliable than self-report; however, a combination of biomarkers/specimens may be clinically useful to evaluate maternal and prenatal exposure [28, 34, 66, 67]. A study from the Helsinki University Hospital Diagnostic Center analyzed 3000 de-identified blood specimens for PEth and identified that 5.2% were positive for PEth at concentration of ≥2 ng/mL, but the positivity rate decreased at higher cutoffs (≥ 8 ng/mL 2.0%, ≥20 ng/mL, 1%), thus lower reporting cut-offs for PEth may be needed [66]. Consequently, the authors supported including PEth in routine prenatal screening [28, 34, 66, 67].

Our study does have key limitations. The patient population tested by ARUP Laboratories consisted of either known alcohol users or patients who must abstain from alcohol use. The laboratory did not have access to the clinical history and pregnancy status of patients from the retrospective data set and residual patient samples from the University of Utah Health. The inclusion criteria for a clinically ordered PEth analysis were unknown; therefore, it is not possible to extrapolate from this test population to the broader population at large. The study for the University of Utah Health patient samples was conducted over a short timeframe, which impacted the number of specimens evaluated. Moreover, an assumption can be made that samples obtained from the University of Utah Health OB/GYN clinical units (general OB/GYN, labor and delivery, emergency OB/GYN services, etc.) would have a higher likelihood of patients being pregnant or wanting to become pregnant compared to samples collected from other non-OB/GYN clinical units (neurology, cardiovascular, internal medicine, etc.). PEth results from the OB/GYN clinical units had a lower PEth positivity rate (1%) than those collected from non-OB/GYN clinical units (9%).

## Conclusion

In summary, PEth is a sensitive and specific biomarker for assessing alcohol exposure in comparison to traditional alcohol biomarkers. Monitoring more than one homologue for PEth may improve the ability to evaluate alcohol abstinence within a timeframe or detect recent alcohol use. Consultation and prenatal screening for alcohol use in individuals who are pregnant or may become pregnant may reduce the incidents of fetal alcohol spectrum disorders. From this study, we hope that OB/GYNs and other clinicians can use this data as an educational tool to prompt discussion about alcohol consumption during pregnancy and FASD with their patients.
